# Syphilis of the Throat and Nose.—II

**Published:** 1908-05-16

**Authors:** 


### Laryngology and Rhinology.

### SYPHILIS OF THE THROAT AND NOSE.—II.

In the fauces, ulceration is the most apparent of the late lesions of syphilis, for infiltrations break down very rapidly in this region and cause little pain while they remain intact. The ulcers are mostly found on the soft palate, faucial pillars, and posterior pharyngeal wall. A very favourite site is the upper surface of the soft palate, and the only visible sign, until the rhinoscope mirror is used, may be an injected, relaxed, and swollen condition of the palate; perforation of the palate may then take place with extraordinary rapidity. Ulcers are rounded and punched out with sharply cut hyperæmic margins and flat yellowish bases. The uvula and the entire soft palate may be destroyed, and perforation of the hard palate is also found, and is pathognomonic of syphilis. Phagedenic ulceration occasionally occurs in broken-down and alcoholic subjects, and may prove fatal.

Isolated gummata are rarely seen, for they break down and ulcerate very quickly; they are, however, occasionally found on the palate, tonsil, or posterior wall of the pharynx. The ulceration heals with a large amount of dense fibrous tissue; stellate scars on the palate and pharynx are very characteristic. Frequently the soft palate becomes partly adherent to the back of the pharynx, or even completely so, occluding the naso-pharynx; less often a cicatricial web forms lower down behind the base of the tongue and interferes with deglutition.

### Syphilis of the Larynx.

In the larynx syphilis may attack any part: commonly the lesions have the appearance of spreading thither from the faucial region, which is usually affected before the larynx. Thus the epiglottis is the most frequently and severely attacked, and on the whole the anterior parts of the larynx are the most affected. This is in marked contradistinction to tuberculosis, which attacks the posterior regions of the larynx in preference to the anterior, and the interior of the organ before it invades the upper aperture. Diffuse infiltration causes tumefaction, which later breaks down into superficial ulcers; the infiltrated parts are dusky red in colour. The epiglottis becomes swollen and variously contorted; serpiginous ulcers appear, and the entire part is frequently destroyed. On the vocal cords a red nodular infiltration is not uncommon; the front half of the cord is often most affected, and not infrequently one cord is diseased while its fellow remains normal or only slightly involved. Thickening in the anterior commissure is common, and the cords may become adherent in front. Subglottic infiltration is often found, and the disease may spread far down in the trachea; abduction of the cords is frequently limited, and stenosis at the level of the glottis is much commoner than in tuberculous laryngitis.

Circumscribed gummata are rare in the larynx, and attack usually the epiglottis, arytenoid or lateral wall. A gumma is generally associated with diffuse infiltration of other parts of the larynx, and is nearly always single and unilateral. It forms a smooth rounded mass of a deep red colour, and tends to break down and ulcerate rapidly. Rarely, however, it appears to undergo a fibrotic process, and persists without change over a prolonged period. Syphilitic lesions give a general impression of firmness, definiteness and solidity, in marked contrast to the soft and ill-defined tumefactions and ulcerations of tuberculosis. On healing there is marked fibrosis producing deformity and distortion of the parts. Stenosis of the larynx follows in severe cases, especially in the neighbourhood of the glottis, more rarely at the upper aperture.

The pain resulting from syphilitic lesions in the fauces and larynx is generally slight, and this is of diagnostic value. But in this connection a warning must be given; the painfulness of a lesion should not be considered an argument against its syphilitic origin, for not infrequently syphilitic ulcers and gummata of the fauces and, more especially, of the upper aperture of the larynx are most intensely painful. The ordinary raucous voice of syphilitic laryngitis is proverbial, and as different as possible from the weak aphonia of tuberculosis.

### Treatment.

The treatment is much the same as that of syphilis generally. The late lesions of the fauces and larynx are often of great severity. In all but the mild and early forms iodides and mercury should be administered together. Potassium iodide must be given in sufficient doses, from thirty grains a day; in severe cases a daily dosage of ninety grains and upwards is required. The larger doses must be taken well diluted in water, or, better, in a tumblerful of milk. For ordinary cases the mercury may be administered by the mouth in any of the usual forms with satisfactory results; but in the rapid, extensive and intractable cases it is essential to get the patient fully under the influence of the drug as quickly as possible, or irreparable damage may be done. For this purpose intramuscular injections are to be recommended; the soluble salts are less painful and less liable to be followed by complications; a 1 per cent. solution of benzoate of mercury with 2 per cent. of sodium benzoate and 2½ per cent. of sodium chloride answers the purpose well; it is injected, in doses of 30 to 50 minims, deeply into the buttock on alternate days. Local specific treatment is usually unnecessary; simple antiseptic douches and sprays are indicated. The nose should be cleared of crusts and an oily spray given to prevent them from re-forming. Condylomata are sometimes very persistent, and the careful application of silver nitrate may be used to hasten their disappearance. Local fumigations of calomel are of great value in foul ulceration of the throat. Laryngeal stenosis may call for tracheotomy, which is to be preferred to intubation as long as active disease is present; no attempt should be made to dilate the stricture until healing is complete.

